# Prolonged grief in African contexts: Scale validation, prevalence rates and risk factors among young adults in Kenya, Namibia and South Africa

**DOI:** 10.1017/gmh.2026.10220

**Published:** 2026-05-18

**Authors:** Clare Killikelly, Daniel V. Hofmann, Stephen Asatsa, Amber Gayle Thalmayer

**Affiliations:** 1 Psychology, University of Zurich, Switzerland; 2 The Catholic University of Eastern Africa, Kenya; 3 United States International University, Africa; 4 University of the Free State, South Africa

**Keywords:** prolonged grief disorder, psychometric validation, prevalence rates, risk factors, Africa

## Abstract

Although grief is a universal part of the human experience, for some, it may become a debilitating mental health disorder. Prolonged grief disorder (PGD) has recently been included in diagnostic guidelines worldwide, but there is little research on global applicability. To our knowledge, this is one of the preliminary studies to examine PGD rates, risk factors and psychometric validity of the International Prolonged Grief Disorder Scale (IPGDS) concurrently across multiple African countries. It is also one of the first studies to examine probable PGD in young adults anywhere, and importantly, in a context where bereavement is often experienced earlier and more often. Psychometric validity and reliability of the IPGDS were confirmed, and exploratory factor analysis supported a one-factor structure. Despite significantly higher exposure to loss and death than in the Western samples, prevalence rates for probable PGD were similar (Kenya 9.63%, South Africa 6.85% and Namibia 5.34%, vs. the global average of 9.8%). Risk factors identified in all three samples include a close relationship to the deceased and a violent death. Gender differences were seen in Kenya and Namibia, with higher rates of probable PGD for women. Financial difficulties increased disorder-level risk in Kenya and South Africa.

## Impact statement

This study makes a timely and important contribution to global mental health by advancing the evidence base on Prolonged Grief Disorder (PGD) beyond high-income, Western contexts. By examining prevalence, risk factors, and the psychometric performance of the International Prolonged Grief Disorder Scale (IPGDS) across Kenya, South Africa and Namibia, it addresses a critical gap in culturally diverse validation research. The confirmation of a robust one-factor structure and strong reliability supports the IPGDS as a viable tool for cross-cultural assessment, enabling more equitable identification of those in need of care. Notably, the study challenges assumptions that higher exposure to bereavement necessarily translates into higher disorder prevalence. Despite frequent and often early-life loss, PGD rates were comparable to global averages, pointing to potential protective mechanisms embedded in social, cultural and religious practices. At the same time, the identification of consistent risk factors – such as violent loss, close kinship and financial strain – highlights targets for intervention and prevention strategies. By focusing on young adults, an under-researched population in grief research, this work provides novel insights into early-life vulnerability and resilience. Overall, these findings inform culturally sensitive diagnostic practices, guide resource allocation and underscore the importance of integrating local strengths into global mental health frameworks.

## Introduction

Grief is a universal part of the human experience, but for some, it may become a debilitating, devastating mental health disorder (Killikelly and Maercker, 2017). Prolonged grief disorder (PGD) is a new mental health disorder included in the World Health Organization (WHO) international classification of diseases, 11th edition (ICD-11) in 2018 and the diagnostic and statistical manual of mental disorders (DSM-5-TR) diagnostic guidelines in 2022. PGD is defined by core symptoms such as yearning and preoccupation with the deceased, symptoms of emotional pain, impairment in functioning, with duration and intensity *markedly exceeding expected social, cultural or religious norms for the individual’s culture and context* (World Health Organization, 2020). This last diagnostic item, “the cultural caveat,” presents a challenge for clinicians and researchers. Presently, there are no clear guidelines for how to assess the relevant cultural norms related to grief accurately and reliably, and/or their influence on grief symptoms. For clinicians working in multi-cultural settings, this can lead to misdiagnosis, difficulty establishing rapport and poor treatment outcomes (Aggarwal et al., 2014). The role of context is also relevant because the frequency of untimely deaths, including infant mortality, varies greatly across contexts (Gang et al., 2023; Wangou et al., 2023). Finally, some societies afford more access to rituals, traditions and support networks that may serve as protective factors and enhance resilience. Fortunately, new research is emerging from around the world to examine the prevalence of PGD in different contexts and explore unique presentations of grief symptoms (i.e., cultural concepts of distress) (Lewis-Fernández and Kirmayer, 2019). In this study, we build on these findings by establishing the psychometric validity of the International Prolonged Grief Disorder Scale (IPGDS) among young adults in South Africa, Namibia and Kenya. To our knowledge, this is one of the first studies to examine prevalence rates of PGD and possible risk factors that may predict grief symptoms across multiple African countries. In addition, to our knowledge, this is the first study to examine probable rates of PGD in a young adult sample anywhere in the world. Documentation of probable PGD rates is particularly important in African contexts, as individuals may experience higher rates of death early in life (Macedo *et al.* 2018).

### Prolonged grief disorder across contexts

Previous research has examined probable rates of PGD in children and adolescents. A recent systematic review identified five studies of children 18 and under (PGD rates between 10% and 32%), but none with a young adult sample (i.e., 18–25) (Falala et al., 2024). Although the present study targets young adults, analogous research has investigated comparable age cohorts in varied contexts, exemplified by Bryant et al. (2021), who examined prolonged grief symptoms among young adults in refugee populations. Children of adults with probable PGD were more likely to have higher mental distress. The current study is the first in the field to examine probable PGD in a young adult sample. Research on PGD has been very active in Western (i.e., North American, European and Australian) and Asian contexts. Systematic reviews and meta-analyses pool prevalence rates at approximately 10% from samples in Europe, North America and China (Lundorff et al., 2017; Rosner et al., 2021). However, this rate varies based on the type of measure used as well as the population sampled (Stelzer et al., 2020). Recently, Comtesse et al. (2024) conducted a cross-national analysis of the prevalence of PGD on the first data available from African and Middle Eastern samples, including Ghana, Togo, Kenya, Turkey and Nigeria. The cross-national point prevalence was found to be 13% (i.e., people from the general population who currently meet PGD diagnostic criteria), indicating that PGD is a notable issue and is prevalent around the world.

Only a handful of studies have explored grief or disordered grief in Africa, and none in young adults. In 2020, the first screening for ICD-11-based psychiatric disorders (adjustment disorder, post-traumatic stress disorder (PTSD) and complex PTSD, and PGD) took place in Kenya, Nigeria and Ghana (Ben-Ezra et al., 2020). This was the first study to systematically assess these disorders, including PGD, in these countries. The prevalence rate for probable PGD was 3.7%. Interestingly, the rate for PGD was much lower than the cross-national prevalence (13%), although 61.6% of the sample indicated that the loss of a loved one was a stressful life event. This indicates the need for in-depth examination of protective factors in African contexts, for example, the highly collectivist nature of most African societies (Adjei, 2019), which is considered a buffer against adversity (Drury et al., 2009). While the suggestion that lower observed prevalence rates of PGD in certain populations may reflect protective factors, this explanation is overly narrow. Alternative factors, including cultural differences in grief expression, limitations of existing measurement tools and the potential mismatch of Western-based diagnostic criteria with local mourning practices, may also contribute to these findings.

African leaders in global health have called for increased research initiatives to track the prevalence rates of mental health disorders, access to care and policy implementation in Africa (Sankoh et al., 2018). Improved reporting would help guide resources and funding to target the most pressing mental health needs in these countries. Information on rates of trauma and PGD is urgently needed. To our knowledge, this is the first study to systematically assess PGD prevalence in these three countries. Examining PGD in African contexts is particularly important due to the disproportionately high exposure to bereavement resulting from infectious diseases, conflict, and limited access to healthcare (World Health Organization, 2023, World Health Statistics Report), combined with the underrepresentation of African populations in PGD research. Existing diagnostic frameworks are predominantly derived from Western samples and may fail to capture culturally specific mourning practices and symptom expressions, underscoring the need for contextually informed epidemiological studies to guide the development of culturally sensitive assessments and interventions (Killikelly and Maercker, 2017).

### The International Prolonged Grief Disorder Scale

Although previous validated assessment measures of PGD exist (i.e., PG-13 scale and the Traumatic Grief Inventory Self Report +), the IPGDS offers a unique assessment tool that balances the need for a universally reliable measure of PGD and the need to contextually assess cultural differences in grief symptoms. The scale contains two parts: one part standard ICD-11 items, and the second part is a catalog of culturally relevant symptoms. This two-part measure was previously validated in diverse socio-cultural contexts, including China, Japan, Switzerland, Turkey, Iran and Israel (Killikelly et al., 2020, 2023). Part two adds to the standard diagnosis by indicating potential culturally relevant symptoms for treatment planning (Killikelly and Maercker, 2023). For example, qualitative interviews with Syrian refugees indicated that items related to somatic distress, anger and religious beliefs should be added to the IPGDS to improve relevance for this group (https://pubmed.ncbi.nlm.nih.gov/40177334/). The current study explores the psychometric validity of the two-part IPGDS in African countries. This will refine current understandings of the measure and disorder with a test of its cross-cultural relevance and provide local clinicians with a valid measure for PGD assessment.

### Risk factors and correlates for probable prolonged grief

Various social, cultural and ecological contextual factors have been found to amplify grief severity (Heeke et al. 2017; Hilberdink et al. 2023). For example, in Togolese and French samples, no significant differences in PGD rates were found (Kokou-Kpolou et al., 2017). However, they identified being male, highly educated and more recently bereaved as risk factors in the African but not in the French sample. Similarly, in an Arab and African sample, Lechner-Meichsner and Comtesse (2022) identified lack of social support, close relationship to the deceased and inability to participate in rituals as specific risk factors for PGD. In South African adolescents, Thurman et al. (2018) reported loss of a biological parent and economic stressors as key determinants of complicated grief. Among Kenyan orphans, a grief prevalence of 66% was reported, but they were not screened for PGD (Ngesa et al., 2019).

PGD prevalence in African populations may be strongly influenced by culturally specific perspectives on death and bereavement. In many African contexts, the bereaved maintain ongoing bonds with the deceased through beliefs about the active role of ancestors in daily life and through extended mourning rituals Such rituals, which facilitate continuing bonds, are theorized to support adaptive grief processes by preserving attachment ties in a culturally meaningful manner (Stroebe et al., 2007; Klass and Steffen, 2018). Research on attachment and continuing bonds suggests that maintaining symbolic or spiritual connections with the deceased can provide emotional regulation, social support and a sense of purpose, potentially serving as a protective factor against the development of PGD (Klass and Steffen, 2018). Therefore, investigating PGD within African contexts requires careful consideration of these culturally embedded practices, as conventional Western diagnostic frameworks may not fully capture the adaptive functions of ongoing attachment to the deceased (Makgahlela, 2016; Asatsa, 2020; Sodi et al., 2021; Hewson et al., 2023). In many African communities, traditional grief rituals remain an important part of the grieving process, serving as a connection between the living and the dead and facilitating psychological transition to a post-grief state (Romanoff and Terenzio, 1998). Zhang and Jia (2020) report that rituals that are performed soon after the loss and on special days lower PTSD, anxiety and depression symptoms.

Emphasis on the role of ancestors in daily life, often in terms of policing proper behavior, is part of traditional beliefs all over the world, and is naturally intertwined with the importance of respect for elders and for kinship ties. In Africa, ancestors are considered part of the members of the family (Awolalu, 1979). Such beliefs are part of ubuntu values (Sodi et al., 2021), and ancestors are believed by many groups to influence fortune and long life or bad luck and illness, as well as future generations through inherited behaviors (Makgahlela, 2016). Ancestors are believed to hold superhuman powers that can be accessed by the living for their benefit (Sarpong, 1974).

To inform the nascent study of PGD with information from African contexts, and to support public health in Africa, where death of loved ones before old age is much more common (Hosegood et al. 2004), here we aim to: (1) confirm the psychometric validity of the IPGDS among young adults in three African countries (Kenya, Namibia and South Africa), (2) examine the probable prevalence rates for PGD in these countries, and (3) explore predictors and risk factors for PGD severity within and between the three countries.

## Methods

We used data from the Africa Long Life Study (ALLS; osf.io/7pmh4/), a longitudinal study with ten measurement occasions over 5 years. A pre-registered analysis plan, anonymized data and R scripts for running the analyses are available on the Open Science Framework (https://osf.io/rqw5y/?view_only=18b0e9ca8abd48cdb6d847a8682eae7b), including sufficient information for an independent researcher to reproduce all reported results and methodology. Ethical approval was obtained as a whole by the partner universities, specifically, the Catholic University of Eastern Africa, the University of Namibia, the University of the Free State, the University of the Witwatersrand and the University of the Western Cape, as well as at the national level in Namibia and at the national and county levels in Kenya. For a list of ethical review boards and approval numbers, please see the Supplementary Material. Annual ethical review is mandatory in Kenya, requiring in-person visits to offices in each county. In Namibia and South Africa, authorization from regional counselors and educational officers was obtained prior to recruitment. All methods were performed in accordance with the above-described relevant guidelines and regulations.

### Procedure

Although the larger study includes eight waves, grief was assessed only at Wave 4 to minimize attrition and participant burden, and analyses are therefore based solely on Wave 4 data. The present study uses data primarily from Wave 4 of the ALLS. Young adults were recruited into the ALLS in 2022 or early 2023 from communities throughout Namibia, Kenya and South Africa, including urban and rural areas. Informed consent was obtained at study entry in line with ethical approval standards. At later waves, approximately a third of participants respond to online surveys sent directly from the University of Zurich. The remainder are found on-site by local research assistants who provide a Samsung tablet for completing the survey. Each survey includes approximately 150 items (taking 20–40 min to complete) on personality traits, mental health, values, views and life experiences. More details are available in (Thalmayer et al., 2024).

### Participants

A total of 3,027 participants completed the full survey; these data are drawn from mid-2023. The sample includes speakers of over 50 different languages across the three countries; however, the questionnaires were completed in English. English is the language of secondary education in Kenya, Namibia and South Africa, and all ALLS participants received formal education in English, giving them the literacy required to respond to survey items themselves. Approximately 97% of participants reported receiving formal education in English, and the remaining ~3% were excluded in sensitivity analyses (see Thalmayer et al., 2024). Use of English rather than translations allowed for broader participation across home language groups in these diverse countries, and also allowed for the privacy of a written survey, since many participants primarily learned to read and write in English, not in their home language.

Screening items early in the survey asked if a participant had lost a loved one in their lifetime, and if yes, how many. Those who answered yes to the first saw the IPGDS, described below, and only their data are analyzed here. (Participants who said they did not lose a loved one instead saw alternative measures about cultural mindset.) The analytic sample for the current study includes a total of 1,730 participants (Kenya = 629, Namibia = 586, South Africa = 515, 58.7% female, at the time of survey *M*_age_ = 19.8 years *SD_age_* = .95). See Table 1 for demographic details.

**Table 1.** Demographic characteristics of participants by country Table 1. long description.

Variable	Kenya		Namibia		South Africa	
	*n*	%	*n*	%	*n*	%
Gender						
Female	304	48.87	330	56.99	362	73.13
Male	317	50.96	249	43.01	130	26.26
Other	1	0.16	0	0.00	3	0.61
Financial problems (Wave 3)						
No financial problems	40	6.70	41	7.23	46	11.19
Sometimes problems	375	62.81	318	56.08	282	68.61
Great problems	182	30.49	208	36.68	83	20.19
Employment status (Wave 4)						
Unemployed	117	18.78	101	17.41	59	11.90
Full-time learner/student	414	66.45	444	76.55	395	79.64
Studying and working	50	8.03	21	3.62	30	6.05
Occasional paid work	15	2.41	2	0.34	0	0.00
Regular part-time paid work	13	2.09	5	0.86	6	1.21
Regular full-time paid work	14	2.25	7	1.21	6	1.21

*Note: n* = number of participants in sample, % of the sample.

### Materials

#### The International ICD-11 Prolonged Grief Disorder Scale (IPGDS)

Prolonged grief symptoms were evaluated through the ICD-11 Prolonged Grief Disorder Scale (IPGDS) (Killikelly et al., 2020), including 20 total items in three conceptual sections (See Supplementary Table S1 for an overview). Participants first complete two screening questions to assess the timing and cultural context: (1) When did the loss occur? (less than 6 months ago; 6–12 months ago; 1–5 years ago; 5–10 years ago; 10–20 years ago; more than 20 years ago). (2) My grief would be considered worse (e.g., more intense, severe and/or of longer duration) than for others from my community or culture (yes/no). Participants are then requested to indicate for the 13 items of the standard scale (IPGDS-13) how often they had felt preoccupation, yearning, and symptoms of emotional distress over the past week because of the loss of a loved one, using a 5-point scale: almost never (less than once a month), rarely (monthly), sometimes (weekly), often (daily) and always (several times a day). For consistency with previous studies, we conducted all analyses for the standard scale, including the 13 items plus item 14, the cultural caveat. Finally, we added a subsample of six of 19 original cultural-supplement items (to reduce participant burden), based on informant interviews and validated in German and Chinese samples (Stelzer et al., 2020). The IPGDS was confirmed to be psychometrically reliable and valid with strong internal consistency (Cronbach’s *α* = .92), and high concurrent, divergent and content validity across multiple international samples (see Killikelly et al., 2020, 2023).

#### The International Mental Health Assessment (IMHA)

The IMHA is a 54-item measure of common mental health problems, which can be scored as lower-level subscales or higher-order spectra (Thalmayer et al., 2023). We here use the three most commonly assessed co-morbid subscales for PGD, Depression (8 items), Anxiety (8), Post-Traumatic Stress (PTS, 6), and the *P*-factor, which aggregates those along with Substance Abuse (6), Anger (6). Additionally, IMHA items are used to calculate an index of risk factors by summing all the events from relevant items on the PTS, life stress, sleep issues, anger and interpersonal and relationship conflict scales.

#### Satisfaction with Life Scale (SWLS)

A three-item assessment of satisfaction with life was administered at Wave 4 with answer options ranging from strongly disagree (1) to strongly agree (7). We calculate the sum score for all complete rows.

#### General Self-Rated Health (GSRH)

A single-item measure to assess the self-reported overall health, ranging from poor (1) to excellent (5).

### Analyses

#### Psychometric properties

All analyses were conducted in R. The psychometric validity of the standard IPGDS, the cultural supplement, and the combined scale in each sample was assessed in terms of reliability (Cronbach’s alpha equal or greater than .8) and unidimensionality (McDonald’s hierarchical omega). The structure of the IPGDS was assessed in terms of whether eigenvalues from exploratory factor analysis (EFA) on each of the three versions indicate unidimensionality (second eigenvalues below 1 and EFA item factor loadings below .30). Convergent validity is examined using zero-order correlations between the IPGDS and IMHA subscales for anxiety, depression, trauma and *p*-factor with moderate to strong positive correlations (i.e.,>.25 for moderate and >.50 for strong) expected in all cases. Content validity is assessed using *t*-tests, hypothesizing that grief severity measured by the IPGDS is higher among participants who have experienced significant losses, such as the loss of a close person, a child, or a sudden and violent loss. Discriminant validity is evaluated using zero-order correlations between the IPGDS and measures of general self-reported health and satisfaction with life, expecting weak to moderate negative correlations between grief severity and these measures.

#### Diagnosis algorithm testing

Along with the sum score on the IPGDS, the prevalence is calculated. As an exploratory analysis, three different diagnostic algorithms for probable caseness will be examined (Killikelly et al., 2020). *Strict* criteria for prolonged bereavement require that items 1 or 2, and at least one item from items 3–12, and item 13 are rated with a score ≥4 points (Killikelly et al., 2021). Moderate criteria require that items 1 or 2, and at least one item between 3 and 12, and item 13 are rated with a score ≥3 points. Finally, the Maciejewski et al. (2016) criteria require that items 1 or 2 and three to five items between 3 and 12 are rated with a score ≥4 points. Diagnostic algorithm exploration and testing are recommended at this stage, given the lack of consensus on the exact PGD diagnostic thresholds from the ICD-11. Prevalence rates for PGD for each of the three diagnostic criteria listed in hypothesis 2 will be calculated for each sample. We explore and report the PGD point prevalence for different demographic information (Table 2).

**Table 2.** Diagnostic algorithms for ICD-11 PGD Table 2. long description.

ICD-11 criterion	Strict criteria	Moderate criteria	Maciejewski criteria
**Core symptoms**	At least **1** core symptom (longing or preoccupation) rated **4 (often)** or higher	At least **1** core symptom rated **3 (sometimes)** or higher	At least **1** core symptom rated **4 (often)** or higher
**Emotional distress**	At least **1** emotional distress symptom rated **4 (often)** or higher	At least **1** emotional distress symptom rated **3 (sometimes)** or higher	At least **3** emotional distress symptoms, each rated **4 (often)** or higher

#### Risk factors

We test a linear regression model with several person-specific, socio-demographic and loss-related (i.e., type of loss) predictors and the IPGDS sum score as outcome to investigate correlates of grief symptomatology.

## Results

### Loss experiences

The percentage of the sample who reported loss of a close loved one ranged from 63.4% in Kenya to 52.6% in South Africa and 55.5% in Namibia. The number of losses experienced by each participant ranged from 1 to 10, with two losses slightly more common than a single loss (see Supplementary Table S2 for more detail). The time since the death was most commonly 1–5 years ago (42.6% of combined samples), with substantial portions having experienced a loss more recently (33.4%) and 24% longer than 5 years ago (details by country are in Supplementary Table S3). The most common relationship to the deceased was a grandparent (40.6%, again SSA total), but 19.2% lost a parent, 10.6% a sibling, 4.2% a romantic partner and 1.9% a child (details by country are in Supplementary Table S4). The cause of death was coded as violent (accident, suicide or homicide) from 11.9% of cases in South Africa to 20.3% in Namibia (17.8% in Kenya), with the remainder coded as non-violent (natural cause, substance abuse, COVID-19; answers that described several causes were not included here). When considering time since loss, we compared participants bereaved less than 6 months to those bereaved 6 months or longer (i.e., 6–12 months, 1–5 years, 5–10 years and > 10 years). Participants bereaved less than 6 months had higher grief severity (*M* = 34.8) than those bereaved 6 months or longer (*M* = 29.9), *p* < .001 (Table 3).

**Table 3.** Mean age and education (Years) by country Table 3. long description.

Variable	Kenya *M* (SD)	Namibia *M* (SD)	South Africa *M* (SD)
Age (Wave 4)	19.75 (1.04)	19.74 (0.90)	19.94 (0.81)
Education (years, W4)	12.52 (2.25)	10.52 (2.48)	12.25 (2.08)

*Note: M*: Mean, SD: Standard Deviation. Education was reported at the highest level of education reached and then recoded to years of education.

### Psychometric properties

The second eigenvalues of the EFA results on the standard PGD scale were only slightly above one for each country, and the whole sample (Supplementary Table S5), and item loadings were above .40 in all samples (Supplementary Table S6). Given our interest in a unidimensional construct, we retained the scale as a single factor (a two-factor EFA of the PGD standard scale is provided in Supplementary Table S7). Previous EFA studies have confirmed the predominantly unidimensional structure of the standard scale (core symptoms and associated grief items) (Killikelly et al. 2020). This is also replicated across different cultural groups. The cultural supplement, however, often yields a multifactor solution (Killikelly et al., 2020). Presently, the cultural supplement scale had second eigenvalues below 1 within and across countries and item loadings above .40 (Supplementary Tables S5 and S8). The combined PGD scale had second and third eigenvalues over 1 in the combined sample, Namibia and South Africa (Supplementary Tables S5 and S9), but item loadings in the one-factor solution were over .50 for all items and this model was retained (Supplementary Table S10).

The internal reliability of the standard, cultural and combined scales ranged from moderate to very good with Cronbach’s alpha >.80 and the McDonald’s omega >.68 (see Table 4). Convergent validity associations with the standard, cultural and combined scales are slightly lower than anticipated, especially in Namibia and for Depression and Anxiety (Tables 5 and 6). *T*-tests assessing PGD scale scores for type of relationship and cause of death revealed significantly higher scores in the case of a violent death and a closer relationship, as predicted (Table 7).

**Table 4.** Reliability of standard, cultural and combined scales Table 4. long description.

	Standard scale		Cultural scale		Combined scale	
	α	ωh	α	ωh	α	ωh
SSA total	.93	.83	.87	.76	.95	.83
Kenya	.94	.82	.89	.77	.95	.86
Namibia	.91	.76	.85	.68	.93	.79
South Africa	.94	.82	.86	.74	.95	.83

*Note:* ωh = Omega hierarchical.

**Table 5.** Convergent and discriminant validity of three versions of the IPGDS Table 5. long description.

Measure	SSA Total			Namibia			Kenya			South Africa		
	S	C	B	S	C	B	S	C	B	S	C	B
Depression	.20	.21	.21	.18	.19	.19	.18	.19	.2	.28	.22	.28
Anxiety	.24	.22	.24	.24	.23	.25	.22	.2	.23	.29	.23	.29
PTS	.27	.23	.27	.24	.23	.25	.28	.25	.28	.32	.23	.31
P-Factor	.26	.26	.28	.21	.23	.23	.27	.26	.28	.34	.29	.34
SWLS	−.02	−.02	−.02	−.05	−.05	−.05	.06	.05	.06	−.10	−.08	−.10
GSRH	−.07	−.09	−.08	−.05	−.08	−.06	−.14	−.12	−.14	−.04	−.06	−.05

*Note:* S = standard scale; C = cultural supplement; B = both scales combined.

**Table 6.** R^2^ of the external validity of the scales Table 6. long description.

Measure	SSA_S	SSA_C	SSA_B	Namibia_S	Namibia_C	Namibia_B	Kenya_S	Kenya_C	Kenya_B	SA_S	SA_C	SA_B
Depression	.04	.04	.04	.03	.04	.04	.03	.04	.04	.08	.05	.08
Anxiety	.06	.05	.06	.06	.05	.06	.05	.04	.05	.08	.05	.08
PTS	.07	.05	.07	.06	.05	.06	.08	.06	.08	.10	.05	.10
P-Factor	.07	.07	.08	.04	.05	.05	.07	.07	.08	.12	.08	.12
SWLS	.00	.00	.00	.00	.00	.00	.00	.00	.00	.01	.01	.01
GSRH	.00	.01	.01	.00	.01	.00	.02	.01	.02	.00	.00	.00

**Table 7.** IPGDS scores by type of relationship to deceased and cause of death Table 7. long description.

	SSA total		Kenya		Namibia		South Africa	
	M	SD	M	SD	M	SD	M	SD
	Standard scale (14 items)							
Type of relationship								
Close	34.7*	13.0	35.6**	13.9	34.9*	11.6	33.7**	13.8
Distant	28.7*	11.7	28.5**	12.2	30.2*	10.9	27.2**	11.6
Cause of death								
Non-violent	29.3*	12.0	28.6*	12.1	31.3	11.1	27.6*	12.4
Violent	33.8*	12.7	34.4*	14.2	33.7	11.2	33.3*	13.3
	Cultural supplement (6 items)							
Type of relationship								
Close	14.1**	5.86	13.8**	6.14	14.0*	5.53	14.8**	6.06
Distant	11.3**	5.23	10.7**	5.15	11.9*	5.28	11.4**	5.14
Cause of death								
Non-violent	11.7*	5.39	10.7*	5.06	12.4	5.31	12.0*	5.67
Violent	13.4*	6.00	13.2*	6.70	13.3	5.51	14.3*	5.83
	Combined scale (20 items)							
Type of relationship								
Close	14.1**	5.86	13.8**	6.14	14.0*	5.53	14.8**	6.06
Distant	11.3**	5.23	10.7**	5.15	11.9*	5.28	11.4**	5.14
Cause of death								
Non-violent	41.0*	16.4	39.3*	16.3	43.8	15.4	39.6*	17.1
Violent	47.2*	17.7	47.6*	19.7	47.0	15.7	47.7*	18.6

*Note: M*: Mean, SD: Standard Deviation. **p < .01 and Cohen’s *d* > .5; *p < .05 and Cohen’s *d* > .2; “Close” relationships encompassing spouse or romantic partner, child, sibling and parent, and “Distant” relationships including former spouse or romantic partner, grandparent and other relationships.

### Diagnosis algorithm testing and prevalence rates

The strict and the Maciejewski diagnosis algorithms both showed the same pattern of results and similar values, with Kenya having the highest prevalence (9.63% and 10.3%, respectively), followed by South Africa (6.85% and 8.87%), then Namibia (5.34% and 7.07%). The moderate diagnosis algorithm instead indicated higher values overall and nearly the same prevalence for Kenya (23.8%) and Namibia (23.4%), followed by South Africa (21%). Females had higher prevalence rates on all PGD scales and samples except for the Maciejewski algorithm in Namibia (Table 8). As part of the test for content validity, it was confirmed that those who lost a close person less than 6 months ago had a higher likelihood of probable PGD diagnosis compared to those who lost a close person more than 6 months ago X^2^ (df = 1, *N* = 37) = 12.6, *p* = 0.0003).

**Table 8.** IPGDS prevalence rates in percentage Table 8. long description.

Baseline characteristics	SSA			Kenya			Namibia			South Africa		
	STR	MOD	MAC	STR	MOD	MAC	STR	MOD	MAC	STR	MOD	MAC
Overall	7.34	23.40	8.84	9.63	23.8	10.3	5.34	24.5	7.07	6.85	21	8.87
Female	8.43	25.1	9.74	12.5	26.3	13.2	5.45	26.4	6.97	7.73	22.9	9.39
Male	5.89	20.7	7.47	6.94	21.5	7.57	5.22	21.5	7.57	4.62	16.2	7.69

*Note*: STR: Strict; MOD: Moderate; MAC: Maciejewski.

### Risk factors

Across all three countries, a close relationship to the deceased and a violent death were associated with higher PGD symptom severity, although the latter was not significant in Namibia. Being male was associated with lower PGD scores in Kenya and Namibia, but not in South Africa. Financial difficulties were inconsistently related to PGD: in Kenya and South Africa, experiencing great financial difficulties was a significant risk factor on the standard scale, whereas “sometimes” experiencing financial difficulties was additionally significant in South Africa. In Namibia, financial difficulties were not significantly associated with PGD. Several potential risk factors were non-significant, including violent death in Namibia, gender in South Africa, and financial difficulties (“great” or “sometimes”) in Namibia, as well as “sometimes” financial difficulties in Kenya on all scales except the cultural measure in South Africa. Full results for the standard, cultural and combined PGD scales are presented in Table 9.

**Table 9.** Risk factors for IPGDS symptoms Table 9. long description.

	Standard			Cultural			Combined		
	*β*	*SE*	*t*	*β*	*SE*	*t*	*β*	*SE*	*t*
	Kenya								
(Intercept)	33.045	2.308	14.318***	13.652	.991	13.773***	46.697	3.113	15.000***
Relationship = Distant	−6.016	1.165	−5.164***	−2.502	.500	−5.002***	−8.518	1.571	−5.421***
Cause = Violent	3.734	1.384	2.698**	2.014	.594	3.388***	5.748	1.867	3.079**
Gender = Male	−2.474	1.048	−2.360*	−1.636	.450	−3.633***	−4.11	1.414	−2.906**
Financial Difficulties = Great	5.331	2.254	2.365*	1.154	.968	1.192	6.484	3.040	2.133*
Sometimes	.962	2.141	.449	−0.43	.920	−.467	.532	2.889	.184
	Namibia								
(Intercept)	35.172	1.939	18.136***	14.756	.932	15.841***	49.928	2.687	18.579***
Relationship = Distant	−4.664	.981	−4.753***	−2.112	.471	−4.479***	−6.776	1.360	−4.983***
Cause = Violent	.409	1.208	.339	.250	.580	.430	.659	1.674	.393
Gender = Male	−1.982	.961	−2.062*	−1.09	.462	−2.361*	−3.072	1.332	−2.307*
Financial Difficulties = Great	1.664	1.92	.867	−.560	.922	−.607	1.104	2.661	0.415
Sometimes	−.470	1.86	−.253	−.434	.893	−.486	−.904	2.577	−0.351
	South Africa								
(Intercept)	28.834	2.209	13.056***	12.214	.971	12.581***	41.048	3.017	13.608***
Relationship = Distant	−5.585	1.365	−4.092***	−2.832	.600	−4.721***	−8.417	1.864	−4.515***
Cause = Violent	4.086	1.939	2.107*	1.216	.852	1.426	5.302	2.649	2.002*
Gender = Male	−1.573	1.446	−1.088	−.167	.635	−.262	−1.740	1.974	−.881
Financial Difficulties = Great	4.877	2.384	2.046*	1.861	1.048	1.776	6.738	3.256	2.069*
Sometimes	4.531	2.035	2.227*	2.447	.894	2.736*	6.978	2.779	2.511*

*Note:* **Standard**: Kenya: *F*(5, 558) = 14.09, *p* < .001, with *R* ^2^ = .112 and adjusted *R* ^2^ = .104. Namibia: *F*(5, 534) = 6.867, *p* < .001, with *R* ^2^ = .060 and adjusted R^2^ = .052. South Africa: *F*(5, 383) = 6.605, *p* < .001, with *R* ^2^ = .079 and adjusted R^2^ = .067. **Cultural**: Kenya: *F*(5, 558) = 15.08, *p* < .001, with *R* ^2^ = .119 and adjusted *R* ^2^ = .111. Namibia: *F*(5, 534) = 5.421, *p* < .001, with *R* ^2^ = .048 and adjusted *R* ^2^ = .039. South Africa: *F*(5, 383) = 7.307, *p* < .001, with *R* ^2^ = .087 and adjusted *R* ^2^ = .075. **Combined**: Kenya: *F*(5, 558) = 16.01, *p* < .001, with *R* ^2^ = .125 and adjusted *R* ^2^ = .118. Namibia: *F*(5, 534) = 6.987, *p* < .001, with *R* ^2^ = .061 and adjusted *R* ^2^ = .053. South Africa: *F*(5, 383) = 7.46, *p* < .001, with *R* ^2^ = .089 and adjusted *R* ^2^ = .077. β = unstandardised regression coefficient; SE = standard error; t = t value. Reference categories: close relationship, non-violent death, female, and no financial problems. * p < .05, ** p < .01, *** p < .001.

## Discussion

To our knowledge, this is the first study to find evidence for the psychometric validity of a measure for PGD, to estimate diagnostic rates, and to explore risk factors and correlates for probable disorder in Kenya, Namibia and South Africa. This is the first study in the field to examine probable rates of PGD in young adults. Evidence for the psychometric validity of the IPGDS was confirmed with strong reliability and validity and good support for a one-factor structure. Using the strict diagnostic algorithm, prevalence rates for PGD were found within the expected range (Kenya 9.63%, South Africa 6.85% and Namibia 5.34%). Risk factors identified included a close relationship to the deceased and violent death. Gender differences were found in Kenya and Namibia, while financial difficulties were a risk factor in Kenya and South Africa. We discuss here the psychometric validity of the IPGDS in the African context, particularly, the possible clinical utility of using the cultural supplement. We then present the prevalence rates in comparison with PGD rates worldwide, and finally, an exploration of risk factors and possible explanations for protective mechanisms.

The IPGDS can provide clinicians and researchers with a reliable and valid assessment tool for PGD. Both the standard scale and key items from the cultural supplement were found to be reliable and valid in Kenya, Namibia and South Africa. There is very little research on chronic disorders, predictors of severity and underlying etiological mechanisms following bereavement in contexts outside of Western contexts. Beliefs about mental illness could be qualitatively different and may significantly impact symptom presentation and their relationship to health services. Additionally, individuals have the right to preserve their own cultural belief systems about health (Maltseva, 2018). The cultural supplement of the IPGDS offers additional items that may be particularly relevant in different contexts. For example, in the current sample, the most strongly endorsed item of the cultural supplement, across the whole sample and in each country, was item 4 from the cultural supplement, “I constantly look back upon the past relationship.” Interestingly, the least endorsed was item 3, “The loss shattered my trust in life or faith in God/a higher spiritual power.” This indicates that although participants experienced strong standard symptoms of grief (item 4 is related to core symptom preoccupation), this did not impact their faith. This strong connection to spiritual beliefs may be an important protective factor (Koburtay et al., 2023).

Specific to prolonged grief, cultural groups may have distinct conceptualizations of the disorder and may experience different symptoms or clusters of symptoms. Additionally, cultural variations in the expression of distress, the course of the disorder, and accepted treatment approaches can influence diagnostic accuracy, help-seeking behavior, and the overall recovery process. Recent research on bereaved refugees in sub-Saharan Africa explored culturally grounded explanatory models of PGD. The study found that participants primarily attributed PGD to psychological causes and believed that recovery could be achieved through social and religious support. Psychological or professional interventions were rarely mentioned, consistent with findings from Namibia regarding trauma-related distress. Participants also identified barriers to support, including stigma and cultural misunderstandings. The inclusion of religious beliefs and rituals in therapeutic approaches was highlighted as a key factor for culturally appropriate care, particularly in contexts where conventional psychotherapy may not be widely accepted.(Stroebe and Schut, 1998; Rosenblatt, 2008; Kirmayer, 2012; Claudius et al., 2022; Lechner-Meichsner and Comtesse, 2022) Previous research using core items of the IPGDS and the strict diagnostic algorithm has confirmed similar prevalence rates for PGD worldwide: Switzerland (11.7%), China (13.5%), Israel (2%), Portugal (6.1%) and Ireland (1.3%) (Killikelly et al., 2020, 2021). Variation in prevalence rates may be due to inconsistencies in methodological rigor, sample differences or true differences in the rate of disorder. The rates in Kenya 9.63%, South Africa 6.85% and Namibia 5.34%, are in line with these previous findings, and moreover, below the cross-national average of 13%. The lower rates of PGD in the African contexts may be considered a surprising finding given the high exposure to trauma commonly found in similar contexts (Duckers and Brewin, 2018). In this sample, most participants (74.4%) experienced two or more losses, and 47.7% experienced three or more losses. Rates of trauma exposure were also high, 31.8%. Curiously, previous research has also found relatively low rates of stress-related disorders in African countries. Ben-Ezra et al. (2020) found that rates of adjustment disorder and PGD were lower than in other countries despite high trauma exposure. This may imply the presence of certain resilience and protective factors that may be culturally linked, similar to the “vulnerability paradox” that has been identified with PTS (Duckers and Brewin, 2018).

The interpretation of lower PGD prevalence rates as indicative of protective factors should be approached with caution. While cultural values, social support systems, and collective coping strategies may indeed buffer against grief-related distress, alternative explanations must also be considered (Wojtkowiak et al., 2021). Cultural variations in the expression and communication of grief symptoms can influence how distress is recognized and reported, potentially leading to underestimation of prevalence (Kirmayer et al., 2017). Moreover, measurement limitations – such as the use of screening instruments that have not been culturally validated – may fail to capture culturally specific manifestations of grief. The reliance on diagnostic criteria developed primarily within Western psychiatric frameworks further compounds this issue, as these criteria may not fully align with local understandings of loss, emotion and adaptation (Osborne, 2026).

Although there are a handful of studies examining risk and protective factors outside of Western and North American settings, that is, Colombia and China (Heeke et al., 2015; Yi et al., 2018), to our knowledge, none have been conducted in Africa. For the first time, we have identified risk factors in these three African countries, and perhaps unsurprisingly, these factors are commonly found around the world. For example, across all three countries, this study confirms that a close relationship to the deceased and a violent death are strong predictors of probable PGD. This is consistently found in previous research (Kristensen et al., 2012). However, each country also has unique contextual factors (cultural, economic and political) that should be considered when modeling risk and protective factors for PGD. For example, the findings that female gender was a risk factor across all measures in Kenya and Namibia but not South Africa attest to possible structural differences in the grief reaction and support. This is particularly interesting given a recent exploration of the mourning rituals for South African widows (Mabunda and Ross, 2023). In some South African cultures, it is believed that the late husband becomes a ghost, and he may prevent the widow from remarrying and moving on with life. Therefore, she must participate in certain rituals to prevent suffering and bad luck. For example, “sittings” or *ukuzila* practice entails wearing certain clothes and sitting alone in an empty room. Mabunda and Ross (2023) questioned these practices and asked participants to consider whether their human rights were violated. Conversely, most participants felt that this was a part of their culture and a way to honor their deceased husbands. Kotzé et al. (2012) explored the role of women in mourning rituals in post-apartheid South Africa. Violent and unnatural loss due to political conflict has been part of women’s lives for generations. The authors propose that women’s rights to perform mourning rituals are closely tied to their hopes, values and community solidarity and may act an of protest against modernization (Kotzé et al., 2012).

Financial difficulties were a risk factor for PGD severity in South Africa and Kenya. Previous research has also found financial stress to be a strong risk factor in South African adolescents. Emerging adulthood is a time of vulnerability, particularly for the onset of psychiatric conditions. Ward-Smith screened 733 low economic status adolescents from South African high schools. They found noticeably high rates of anxiety symptoms (56.9%), depressive symptoms (57.7%), PTSD symptoms (31.4%) and risky alcohol use (35.9%) compared to worldwide rates of approximately 20%–34%. The authors attribute commonly found factors such as poverty, violence and trauma as possible risk factors.

These findings attest to the need for accessible, affordable and culturally acceptable psychological assessment and support for grief and PGD. Currently, there are few tailored interventions for grief, and so far, very few for bereaved people from African countries. Emerging evidence from these countries catalyzes the need to include unique contextual factors in the assessment and treatment of psychological distress. For example, future research may explore rituals, beliefs and values that act as protective factors against the development of PGD despite high numbers of losses and trauma exposure.

## Limitations

The study relied on self-report survey data. Self-report questionnaires to assess diagnosis are limited as it requires insight and recall. Clinical interviews should always be used to confirm a definitive diagnosis. This is why we refer to it as a “probable” diagnosis. Future studies may consider triangulating data collection methods to address the weaknesses of a single method. For example, key informant interviews to identify unique idioms of distress, explanatory models of illness and beliefs about treatment can help guide clinicians and improve the assessment process (Kirmayer et al., 2003). The surveys were completed in English, which is the language of High school education, but not necessarily the mother tongue. This could bias the sample and the results toward a Western-based assessment criterion. A limitation of the present study is the absence of a validated measure of PGD (e.g., TGI-SR or PG-13), which restricts the ability to conduct a convergent validity analysis. Several validations of other PGD measures (e.g., PG-13 derivatives, the Traumatic Grief Inventory–Self Report Plus/TGI-SR+) report **mixed results**: many EFAs yield a unidimensional solution for core symptom sets, but some studies find **two factors** (commonly framed as separation/yearning vs. associated cognitive-emotional/behavioral symptoms). This indicates some heterogeneity depending on instrument version, sample and analytic choices. Differences in sample composition, item set, scale length and the lack of invariance testing may bias the EFA results. Given the aim of developing a culturally robust measurement tool, the absence of additional validation analyses (e.g., test–retest reliability and item-level analyses) represents a limitation, and future research should address this by, for example, conducting longitudinal studies to assess temporal stability and performing item response theory analyses across diverse cultural groups.

## Conclusions

The introduction of the new mental health disorder, PGD, presents a unique opportunity: to develop and refine a new mental health assessment measure and model that can be adapted to the cultural context with direct input from intended users for the improved mental health of their communities. The IPGDS is found to be a valid and reliable scale in many countries, and now in Kenya, Namibia and South Africa. Although rates of PGD are low in these samples, there are still individuals who need early psychological support, and the demand for services for young adults is growing.

## Supporting information

10.1017/gmh.2026.10220.sm001 Killikelly et al. supplementary material Killikelly et al. supplementary material

## Data Availability

The data and code used in the current study are available on the OSF link: https://osf.io/d4yv5/?view_only=477df5e8f6a740858d8f8c600d09ea63

## Supplementary material

The supplementary material for this article can be found at http://doi.org/10.1017/gmh.2026.10220.

## Table 1. Long description

Starting from the top, the table lists variables in the first column: Gender, Financial problems (Wave 3), and Employment status (Wave 4). For each variable, data is presented for Kenya, Namibia, and South Africa in adjacent columns labeled n and percent. Under Gender, Kenya has 304 females (48.87 percent), 317 males (50.96 percent), and 1 other (0.16 percent); Namibia has 330 females (56.99 percent), 249 males (43.01 percent), and 0 other (0.00 percent); South Africa has 362 females (73.13 percent), 130 males (26.26 percent), and 3 other (0.61 percent). For Financial problems, Kenya shows 40 with no financial problems (6.70 percent), 375 sometimes problems (62.81 percent), and 182 great problems (30.49 percent); Namibia shows 41 no problems (7.23 percent), 318 sometimes (56.08 percent), 208 great (36.68 percent); South Africa shows 46 no problems (11.19 percent), 282 sometimes (68.61 percent), 83 great (20.19 percent). Employment status in Kenya: 117 unemployed (18.78 percent), 414 full-time learners (66.45 percent), 50 studying and working (8.03 percent), 15 occasional paid work (2.41 percent), 13 regular part-time (2.09 percent), 14 regular full-time (2.25 percent). Namibia: 101 unemployed (17.41 percent), 444 full-time learners (76.55 percent), 21 studying and working (3.62 percent), 2 occasional paid work (0.34 percent), 5 regular part-time (0.86 percent), 7 regular full-time (1.21 percent). South Africa: 59 unemployed (11.90 percent), 395 full-time learners (79.64 percent), 30 studying and working (6.05 percent), 0 occasional paid work (0.00 percent), 6 regular part-time (1.21 percent), 6 regular full-time (1.21 percent). The note clarifies n as number of participants and percent as percentage of the sample.

Navigate back to Table 1..

## Table 2. Long description

The table has four columns: I C D dash 11 criterion, strict criteria, moderate criteria, and Maciejewski criteria. The first row is core symptoms. For strict criteria, at least one core symptom, longing or preoccupation, must be rated four, often, or higher. For moderate criteria, at least one core symptom must be rated three, sometimes, or higher. For Maciejewski criteria, at least one core symptom must be rated four, often, or higher. The second row is emotional distress. For strict criteria, at least one emotional distress symptom must be rated four, often, or higher. For moderate criteria, at least one emotional distress symptom must be rated three, sometimes, or higher. For Maciejewski criteria, at least three emotional distress symptoms, each rated four, often, or higher, are required.

Navigate back to Table 2..

## Table 3. Long description

The table has two rows for variables and three columns for countries. The first row is Age (Wave 4): Kenya mean 19.75, standard deviation 1.04; Namibia mean 19.74, standard deviation 0.90; South Africa mean 19.94, standard deviation 0.81. The second row is Education in years (Wave 4): Kenya mean 12.52, standard deviation 2.25; Namibia mean 10.52, standard deviation 2.48; South Africa mean 12.25, standard deviation 2.08. Means are labeled as M and standard deviations as S D. Education is reported as the highest level reached, recoded to years.

Navigate back to Table 3..

## Table 4. Long description

The table presents reliability coefficients for three scales: standard, cultural, and combined. Each scale has two columns, alpha and omega h. The rows are SSA total, Kenya, Namibia, and South Africa. For SSA total, standard scale alpha is point nine three, omega h is point eight three; cultural scale alpha is point eight seven, omega h is point seven six; combined scale alpha is point nine five, omega h is point eight three. For Kenya, standard scale alpha is point nine four, omega h is point eight two; cultural scale alpha is point eight nine, omega h is point seven seven; combined scale alpha is point nine five, omega h is point eight six. For Namibia, standard scale alpha is point nine one, omega h is point seven six; cultural scale alpha is point eight five, omega h is point six eight; combined scale alpha is point nine three, omega h is point seven nine. For South Africa, standard scale alpha is point nine four, omega h is point eight two; cultural scale alpha is point eight six, omega h is point seven four; combined scale alpha is point nine five, omega h is point eight three. Omega h is defined as omega hierarchical.

Navigate back to Table 4..

## Table 5. Long description

Beginning with the top row, Depression coefficients are S S A Total S point two zero, C point two one, B point two one; Namibia S point one eight, C point one nine, B point one nine; Kenya S point one eight, C point one nine, B point two; South Africa S point two eight, C point two two, B point two eight. Next, Anxiety coefficients are S S A Total S point two four, C point two two, B point two four; Namibia S point two four, C point two three, B point two five; Kenya S point two two, C point two, B point two three; South Africa S point two nine, C point two three, B point two nine. P T S coefficients are S S A Total S point two seven, C point two three, B point two seven; Namibia S point two four, C point two three, B point two five; Kenya S point two eight, C point two five, B point two eight; South Africa S point three two, C point two three, B point three one. P-Factor coefficients are S S A Total S point two six, C point two six, B point two eight; Namibia S point two one, C point two three, B point two three; Kenya S point two seven, C point two six, B point two eight; South Africa S point three four, C point two nine, B point three four. S W L S coefficients are S S A Total S minus point zero two, C minus point zero two, B minus point zero two; Namibia S minus point zero five, C minus point zero five, B minus point zero five; Kenya S point zero six, C point zero five, B point zero six; South Africa S minus point one zero, C minus point zero eight, B minus point one zero. G S R H coefficients are S S A Total S minus point zero seven, C minus point zero nine, B minus point zero eight; Namibia S minus point zero five, C minus point zero eight, B minus point zero six; Kenya S minus point one four, C minus point one two, B minus point one four; South Africa S minus point zero four, C minus point zero six, B minus point zero five. S equals standard scale, C equals cultural supplement, B equals both scales combined.

Navigate back to Table 5..

## Table 6. Long description

The table lists six measures in the first column: Depression, Anxiety, P T S, P-Factor, S W L S, and G S R H. Each row provides R squared values for each measure across twelve columns, grouped by region and subscale. The columns are S S A underscore S, S S A underscore C, S S A underscore B, Namibia underscore S, Namibia underscore C, Namibia underscore B, Kenya underscore S, Kenya underscore C, Kenya underscore B, S A underscore S, S A underscore C, and S A underscore B. For Depression, values range from point zero three to point zero eight, with the highest in S A underscore S and S A underscore B. Anxiety values range from point zero four to point zero eight, with the highest in S A underscore S and S A underscore B. P T S values range from point zero five to point one zero, with the highest in S A underscore S and S A underscore B. P-Factor values range from point zero four to point one two, with the highest in S A underscore S and S A underscore B. S W L S values are point zero zero or point zero one across all columns. G S R H values are point zero zero to point zero two, with the highest in Kenya underscore S and Kenya underscore B. No measure exceeds point one two in any cell.

Navigate back to Table 6..

## Table 7. Long description

Starting from the top, the table is divided into three main sections: standard scale (14 items), cultural supplement (6 items), and combined scale (20 items). Each section is further split into ‘Type of relationship’ and ‘Cause of death’. For ‘Type of relationship’, rows list ‘Close’ and ‘Distant’ with mean (M) and standard deviation (SD) values for SSA total, Kenya, Namibia, and South Africa. For the standard scale, ‘Close’ scores are 34.7 (SD 13.0) for SSA, 35.6 (SD 13.9) for Kenya, 34.9 (SD 11.6) for Namibia, and 33.7 (SD 13.8) for South Africa. ‘Distant’ scores are 28.7 (SD 11.7) for SSA, 28.5 (SD 12.2) for Kenya, 30.2 (SD 10.9) for Namibia, and 27.2 (SD 11.6) for South Africa. For ‘Cause of death’, ‘Non-violent’ scores are 48.9 (SD 17.7) for SSA, 49.3 (SD 18.9) for Kenya, 48.9 (SD 15.9) for Namibia, and 48.5 (SD 18.8) for South Africa. ‘Violent’ scores are 40.0 (SD 16.0) for SSA, 39.2 (SD 16.5) for Kenya, 42.0 (SD 15.2) for Namibia, and 38.6 (SD 15.9) for South Africa. The cultural supplement and combined scale sections repeat this structure, with ‘Close’ and ‘Distant’ relationship scores and ‘Non-violent’ and ‘Violent’ cause of death scores, each with corresponding mean and SD values for all regions. Statistical significance is indicated by asterisks: double for p less than .01 and Cohen’s d greater than .5, single for p less than .05 and Cohen’s d greater than .2. Definitions clarify that ‘Close’ relationships include spouse, romantic partner, child, sibling, and parent, while ‘Distant’ relationships include former spouse, grandparent, and other.

Navigate back to Table 7..

## Table 8. Long description

From left to right, the first column lists baseline characteristics: Overall, Female, and Male. The next twelve columns are grouped by region: S S A, Kenya, Namibia, and South Africa, each with S T R, M O D, and M A C subcategories. For Overall, S S A rates are 7.34 for S T R, 23.40 for M O D, 8.84 for M A C; Kenya rates are 9.63 for S T R, 23.8 for M O D, 10.3 for M A C; Namibia rates are 5.34 for S T R, 24.5 for M O D, 7.07 for M A C; South Africa rates are 6.85 for S T R, 21 for M O D, 8.87 for M A C. For Female, S S A rates are 8.43 for S T R, 25.1 for M O D, 9.74 for M A C; Kenya rates are 12.5 for S T R, 26.3 for M O D, 13.2 for M A C; Namibia rates are 5.45 for S T R, 26.4 for M O D, 6.97 for M A C; South Africa rates are 7.73 for S T R, 22.9 for M O D, 9.39 for M A C. For Male, S S A rates are 5.89 for S T R, 20.7 for M O D, 7.47 for M A C; Kenya rates are 6.94 for S T R, 21.5 for M O D, 7.57 for M A C; Namibia rates are 5.22 for S T R, 21.5 for M O D, 7.57 for M A C; South Africa rates are 4.62 for S T R, 16.2 for M O D, 7.69 for M A C. S T R stands for Strict, M O D for Moderate, and M A C for Maciejewski.

Navigate back to Table 8..

## Table 9. Long description

Starting from the top, the table is divided into three main sections for Kenya, Namibia, and South Africa. Each section contains three model columns: Standard, Cultural, and Combined, with subcolumns for beta, S E, and t values. For Kenya, the intercept values are 33.045 (Standard), 13.652 (Cultural), and 46.697 (Combined), all with highly significant t values. Relationship equals Distant shows negative beta values across all models, with t values indicating strong significance. Cause equals Violent has positive beta values, significant in Standard and Combined models. Gender equals Male yields negative beta values, significant in most models. Financial Difficulties equals Great shows positive beta values, significant in Standard and Combined models. Sometimes is mostly non-significant. Namibia’s intercepts are 35.172 (Standard), 14.756 (Cultural), and 49.928 (Combined), all highly significant. Relationship equals Distant is negative and significant across all models. Cause equals Violent is non-significant. Gender equals Male is negative and significant in all models. Financial Difficulties equals Great is mostly non-significant. Sometimes is non-significant. South Africa’s intercepts are 28.834 (Standard), 12.214 (Cultural), and 41.048 (Combined), all highly significant. Relationship equals Distant is negative and significant. Cause equals Violent is positive and significant in Standard and Combined models. Gender equals Male is negative but mostly non-significant. Financial Difficulties equals Great is positive and significant in Standard and Combined models. Sometimes is positive and significant in all models. Statistical notes below the table provide F, p, R squared, and adjusted R squared values for each country and model.

Navigate back to Table 9..
