# Supplementary material for: Prolonged grief in African contexts: Scale validation, prevalence rates and risk factors among young adults in Kenya, Namibia and South Africa
Source: Glob Ment Health (Camb). 2026 May 18;13:e114. doi: 10.1017/gmh.2026.10220 (PMC13244226; doi:10.1017/gmh.2026.10220)
Supplement: Killikelly et al. supplementary material [file S2054425126102209sup001.docx]

**Supplemental Material**

**Deviations from Pre-registered Analysis Plan**

Several deviations from the pre-registered analysis plan were implemented to address data limitations and improve the robustness of the analysis. The cause of death was recoded into two categories: 'Violent' (comprising accident, suicide, and homicide) and 'Non-violent' (including natural cause, substance abuse, and COVID-19), while cases with multiple causes of death (57 out of 1701) were coded as NA. The type of relationship was also recoded, with 'Close' relationships encompassing spouse or romantic partner, child, sibling, and parent, and 'Distant' relationships including former spouse or romantic partner, grandparent, and other relationships. Due to the low response rate, the gender category 'Other' was coded as NA, resulting in group comparisons being conducted using t-tests rather than ANOVAs, and the effect size measure was changed from η² to Cohen's d. Age was removed from the linear regression analysis because of insufficient data. Furthermore, the gender category 'Other' was excluded from the analysis for the same reason. Item-total correlations and mean inter-item correlations were omitted due to an oversight in the pre-registration, but internal consistency was still assessed using Cronbach's alpha, McDonald's omega, and Exploratory Factor Analysis (EFA) item loadings. Additionally, Omega was updated to Omega hierarchical to better fit the hierarchical structure of the data.

**Supplemental Table S1**

*Item Overview*

| Item Name | Item Content |
| --- | --- |
|  | Standard |
| IPGDS_STAND1 | I am longing or yearning for the deceased. |
| IPGDS_STAND2 | I am preoccupied with thoughts about the deceased or circumstances of the death. |
| IPGDS_STAND3 | I have intense feelings of sorrow, related to the deceased. |
| IPGDS_STAND4 | I feel guilty about the death or circumstances surrounding the death. |
| IPGDS_STAND5 | I am angry over the loss. |
| IPGDS_STAND6 | I try to avoid reminders of the deceased or the death as much as possible (e.g., pictures, memories). |
| IPGDS_STAND7 | I blame others or the circumstances for the death (e.g., a higher power). |
| IPGDS_STAND8 | I have trouble or just don’t want to accept the loss. |
| IPGDS_STAND9 | I feel that I lost a part of myself. |
| IPGDS_STAND10 | I have trouble or have no desire to experience joy or satisfaction. |
| IPGDS_STAND11 | I feel emotionally numb. |
| IPGDS_STAND12 | I have difficulties engaging in activities I enjoyed prior to the death. |
| IPGDS_STAND13 | Grief significantly interferes with my ability to work, socialize or function in everyday life. |
| IPGDS_STAND14 | My grief would be considered worse (e.g., more intense, severe and/or of longer duration) than for others from my community or culture. |
|  | Cultural supplement: Accessory items |
| IPGDS_SUPPL1 | I experience strong physical problems since the loss (e.g., headache, problems with appetite). |
| IPGDS_SUPPL2 | I would do anything to feel close to the deceased (e.g., visit their grave everyday, sleep next to their picture). |
| IPGDS_SUPPL3 | The loss shattered my trust in life or faith in God/a higher spiritual power. |
| IPGDS_SUPPL4 | I constantly look back upon the past relationship. |
| IPGDS_SUPPL5 | I feel he/she is beside me. |
| IPGDS_SUPPL6 | I cry loudly when I think of the loss. |

**Supplemental Table S2**

*Number of Losses Across the Lifetime*

|  | SSA | Kenya | Namibia | South Africa |
| --- | --- | --- | --- | --- |
| 1 | 407 | 174 | 151 | 78 |
| 2 | 425 | 172 | 126 | 121 |
| 3 | 323 | 124 | 101 | 95 |
| 4 | 190 | 64 | 56 | 64 |
| 5 | 124 | 37 | 31 | 54 |
| 6 | 45 | 11 | 11 | 22 |
| 7 | 36 | 3 | 18 | 9 |
| 8 | 23 | 11 | 4 | 8 |
| 9 | 7 | 1 | 1 | 5 |
| 10 | 12 | 4 | 3 | 5 |

*Note. N = 138 NAs were removed.*

**Supplemental Table S3**

*Time of Death Occurrence*

|  | SSA | Kenya | Namibia | South Africa |
| --- | --- | --- | --- | --- |
| Less than 6 months ago | 299 | 102 | 93 | 97 |
| 6 to 12 months ago | 279 | 96 | 91 | 86 |
| 1 to 5 years ago | 737 | 268 | 265 | 193 |
| 5 to 10 years ago | 275 | 100 | 84 | 85 |
| More than 10 years ago | 140 | 57 | 47 | 35 |

**Supplemental Table S4**

*Relationship to Deceased*

| Type of Relationship | SSA | Kenya | Namibia | South Africa |
| --- | --- | --- | --- | --- |
| Spouse or romantic partner | 55 | 16 | 26 | 12 |
| Former spouse or former romantic partner | 18 | 4 | 11 | 3 |
| Child | 33 | 7 | 13 | 13 |
| Sibling | 183 | 57 | 79 | 44 |
| Parent | 330 | 102 | 121 | 101 |
| Grandparent | 703 | 271 | 200 | 216 |
| Other relationship | 408 | 166 | 130 | 107 |

#

**Supplemental Table S5**

# *Eigenvalues of the Standard, Cultural and Combined Scale*

| Factor | Standard | Cultural Supplement | Combined Scale |
| --- | --- | --- | --- |
| SSA | | | |
| 1 | 7.41 | 3.63 | 9.88 |
| 2 | 1.13 | .83 | 1.34 |
| 3 | .81 | .59 | 1.14 |
| 4 | .70 | .51 | .84 |
| 5 | .63 | .44 | .72 |
| 6 | .57 | .00 | .71 |
| Kenya | | | |
| 1 | 7.91 | 3.94 | 1.66 |
| 2 | 1.11 | .72 | 1.44 |
| 3 | .74 | .48 | .97 |
| 4 | .62 | .44 | .76 |
| 5 | .57 | .42 | .73 |
| 6 | .53 | .00 | .63 |
| Namibia | | | |
| 1 | 6.68 | 3.46 | 8.95 |
| 2 | 1.19 | .92 | 1.42 |
| 3 | .98 | .62 | 1.23 |
| 4 | .79 | .57 | 1 |
| 5 | .65 | .44 | .81 |
| 6 | .59 | .00 | .75 |
| South Africa | | | |
| 1 | 7.69 | 3.55 | 1.18 |
| 2 | 1.16 | .85 | 1.3 |
| 3 | .90 | .66 | 1.21 |
| 4 | .67 | .51 | .96 |
| 5 | .58 | .43 | .72 |
| 6 | .54 | .00 | .69 |

**Supplemental Table S6**

# *1-factor EFA of the Standard Scale*

| Item | SSA | Kenya | Namibia | South Africa |
| --- | --- | --- | --- | --- |
| Longing or yearning​ | .64​ | .69 | .61 | .65 |
| Preoccupied with thoughts ​ | .72​ | .76 | .72 | .68 |
| Intense feelings of sorrow​ | .76​ | .78 | .71 | .78 |
| Feel guilty about the death​ | .65​ | .69 | .62 | .62 |
| Angry over the loss​ | .72​ | .75 | .61 | .78 |
| Avoid reminders of the deceased​ | .55​ | .55 | .52 | .56 |
| Blame others for the death ​ | .53 | .61 | .48 | .51 |
| Trouble or just don’t want to accept the loss​ | .75 | .77 | .70 | .78 |
| Feel that I lost a part of myself​ | .71 | .73 | .63 | .78 |
| No desire to experience joy​ | .75​ | .77 | .73 | .76 |
| I feel emotionally numb​ | .74​ | .76 | .73 | .75 |
| Difficulties engaging in activities​ | .76​ | .79 | .71 | .79 |
| Limited ability to function in everyday life​ | .77​ | .78 | .73 | .80 |
| Grief would be considered worse​ | .73​ | .74 | .70 | .75 |

**Supplemental Table S7**

# *2-factor EFA of the Standard Scale*

| Item | SSA |  | Kenya |  | Namibia |  | South Africa |  |
| --- | --- | --- | --- | --- | --- | --- | --- | --- |
|  | MR1 | MR2 | MR1 | MR2 | MR1 | MR2​ | MR1​ | MR2​ |
| Longing or yearning​ | -.09​ | .84 | -.10 | .86 | -.10​ | .81​ | -.06​ | .83​ |
| Preoccupied with thoughts ​ | .00 | .83 | .00 | .84 | .00​ | .83​ | -.03​ | .83​ |
| Intense feelings of sorrow​ | .09 | .77 | .01 | .84 | .13​ | .68​ | .10​ | .81​ |
| Feel guilty about the death​ | .37 | .33 | .21 | .53 | .32​ | .36​ | .48​ | .18​ |
| Angry over the loss​ | .36 | .42 | .27 | .53 | .17​ | .51​ | .48​ | .37​ |
| Avoid reminders of the deceased​ | .29 | .31 | .10 | .49 | .21​ | .36​ | .45​ | .14​ |
| Blame others for the death ​ | .48 | .08 | .61 | .03 | .40​ | .11​ | .39​ | .15​ |
| Trouble or just don’t want to accept the loss​ | .53 | .27 | .47 | .34 | .43 | .33​ | .56​ | .28​ |
| Feel that I lost a part of myself​ | .39 | .38 | .35 | .42 | .33 | .36​ | .39​ | .47​ |
| No desire to experience joy​ | .82 | -.02 | .83 | -.02 | .75​ | .04​ | .80​ | -.01​ |
| I feel emotionally numb​ | .71 | .07 | .71 | .09 | .62​ | .17​ | .75​ | .04​ |
| Difficulties engaging in activities​ | .85 | -.06 | .88 | -.05 | .84​ | -.08​ | .86​ | -.04​ |
| Limited ability to function in everyday life​ | .85 | -.05 | .79 | .03 | .86​ | -.07​ | .92​ | -.09​ |
| Grief would be considered worse​ | .74 | .03 | .80 | -.01 | .64​ | .12​ | .78​ | .01​ |

**Supplemental Table S8**

# *1-factor EFA of the Cultural Supplement*

| Item | SSA | Kenya | Namibia | South Africa |
| --- | --- | --- | --- | --- |
| Strong physical problems since the loss ​ | .65​ | .74​ | .60​ | .62​ |
| Do anything to feel close to the deceased ​ | .67​ | .72​ | .64​ | .65​ |
| Loss shattered my trust in life​ | .62​ | .67​ | .58​ | .60​ |
| Constantly look back upon the past relationship​ | .64​ | .66​ | .63​ | .64​ |
| I feel he/she is beside me​ | .88​ | .90​ | .87​ | .88​ |
| Cry loudly when I think of the loss​ | .88​ | .90​ | .87​ | .88​ |

**Supplemental Table S9**

# *3-factor EFA of the Combined Scale*

| Item | SSA |  |  | Kenya |  |  | Namibia |  |  | South Africa |  |  |
| --- | --- | --- | --- | --- | --- | --- | --- | --- | --- | --- | --- | --- |
| ​ | MR1 | MR2 | MR3 | MR1 | MR2 | MR3 | MR1 | *MR2*​ | *MR3* | *MR1*​ | *MR2*​ | *MR3* |
| Longing or yearning​ | -.09​ | .83​ | .01​ | -.10​ | .84​ | .02​ | -.10​ | .80​ | .00​ | -.09​ | .86​ | .02​ |
| Preoccupied with thoughts ​ | .00​ | .81​ | .04​ | -.04​ | .83​ | .08​ | -.01​ | .84​ | .00​ | -.01​ | .78​ | .03​ |
| Intense feelings of sorrow​ | .09​ | .76​ | .02​ | .05​ | .83​ | -.04​ | .09​ | .65​ | .09​ | .10​ | .80​ | .00​ |
| Feel guilty about the death​ | .39​ | .33​ | -.02​ | .19​ | .51​ | .07​ | .32​ | .37​ | -.01​ | .55​ | .14​ | -.06​ |
| Angry over the loss​ | .33​ | .43​ | .03​ | .26​ | .54​ | .02​ | .11​ | .55​ | .03​ | .45​ | .35​ | .07​ |
| Avoid reminders of the deceased​ | .29​ | .33​ | -.06​ | .12​ | .50​ | -.03​ | .17​ | .43​ | -.07​ | .46​ | .13​ | .00​ |
| Blame others for the death ​ | .48​ | .07​ | .01​ | .56​ | .00​ | .11​ | .42​ | .14​ | -.07​ | .38​ | .11​ | .05​ |
| Trouble or just don’t want to accept the loss​ | .49​ | .29​ | .02​ | .43​ | .35​ | .06​ | .36​ | .38​ | .02​ | .52​ | .28​ | .04​ |
| Feel that I lost a part of myself​ | .32​ | .40​ | .09​ | .30​ | .44​ | .06​ | .24​ | .36​ | .13​ | .32​ | .52​ | .05​ |
| No desire to experience joy​ | .80​ | -.01​ | .00​ | .82​ | .00​ | -.01​ | .72​ | .06​ | .02​ | .79​ | .00​ | .00​ |
| I feel emotionally numb​ | .68​ | .09​ | .03​ | .73​ | .11​ | -.05​ | .53​ | .19​ | .11​ | .74​ | .07​ | -.02​ |
| Difficulties engaging in activities​ | .84​ | -.04​ | .00​ | .84​ | -.01​ | .02​ | .85​ | -.07​ | -.01​ | .85​ | -.01​ | -.02​ |
| Limited ability to function in everyday life​ | .85​ | -.03​ | -.02​ | .79​ | .07​ | -.05​ | .85​ | -.05​ | -.02​ | .89​ | -.08​ | .04​ |
| Grief would be considered worse​ | .75​ | .03​ | -.01​ | .77​ | -.01​ | .04​ | .66​ | .13​ | -.03​ | .78​ | .01​ | -.02​ |
| Strong physical problems since the loss ​ | .51​ | .05​ | .20​ | .40​ | .10​ | .34​ | .47​ | .09​ | .18​ | .59​ | -.02​ | .15​ |
| Do anything to feel close to the deceased ​ | .34​ | .17​ | .26​ | .38​ | .09​ | .32​ | .39​ | .14​ | .21​ | .24​ | .32​ | .21​ |
| Loss shattered my trust in life​ | .59​ | -.09​ | .18​ | .56​ | -.11​ | .26​ | .53​ | -.03​ | .16​ | .60​ | -.07​ | .14​ |
| Constantly look back upon the past relationship​ | .25​ | .28​ | .23​ | .25​ | .27​ | .26​ | .27​ | .29​ | .21​ | .16​ | .41​ | .21​ |
| I feel he/she is beside me​ | .00​ | .00​ | .99​ | .00​ | .01​ | .98​ | .00​ | .00​ | .99​ | .00​ | .00​ | .99​ |
| Cry loudly when I think of the loss​ | .00​ | .00​ | .99​ | .00​ | .01​ | .98​ | .00​ | .00​ | .99​ | .00​ | .00​ | .99​ |

**Supplemental Table S10**

# *1-factor EFA of the Combined Scale*

|  | SSA | Kenya | Namibia | South Africa |
| --- | --- | --- | --- | --- |
| Longing or yearning​ | .62​ | .66​ | .59​ | .64​ |
| Preoccupied with thoughts ​ | .71​ | .74​ | .70​ | .67​ |
| Intense feelings of sorrow​ | .73​ | .74​ | .71​ | .76​ |
| Feel guilty about the death​ | .63​ | .68​ | .61​ | .60​ |
| Angry over the loss​ | .71​ | .73​ | .59​ | .78​ |
| Avoid reminders of the deceased​ | .52​ | .53​ | .48​ | .54​ |
| Blame others for the death ​ | .53​ | .62​ | .46​ | .50​ |
| Trouble or just don’t want to accept the loss​ | .74​ | .76​ | .68​ | .77​ |
| Feel that I lost a part of myself​ | .71​ | .72​ | .63​ | .78​ |
| No desire to experience joy​ | .75​ | .77​ | .73​ | .75​ |
| I feel emotionally numb​ | .75​ | .76​ | .74​ | .75​ |
| Difficulties engaging in activities​ | .76​ | .80​ | .71​ | .78​ |
| Limited ability to function in everyday life​ | .76​ | .77​ | .73​ | .80​ |
| Grief would be considered worse​ | .73​ | .75​ | .69​ | .74​ |
| Strong physical problems since the loss ​ | .68​ | .73​ | .64​ | .65​ |
| Do anything to feel close to the deceased ​ | .66​ | .69​ | .64​ | .66​ |
| Loss shattered my trust in life​ | .61​ | .63​ | .58​ | .62​ |
| Constantly look back upon the past relationship​ | .65​ | .67​ | .65​ | .65​ |
| I feel he/she is beside me​ | .69​ | .74​ | .66​ | .69​ |
| Cry loudly when I think of the loss​ | .69​ | .74​ | .66​ | .69​ |

**Supplemental Table 11**

*Risk Factors for Standard IPGDS Symptoms Across Countries*

|  | Standard | | | Cultural | | | | Combined | | | | |
| --- | --- | --- | --- | --- | --- | --- | --- | --- | --- | --- | --- | --- |
|  | β | *SE* | *t* | | β | *SE* | *t* | β | | *SE* | *t* |  |
|  | SSA | | | | | | | | | | | |
| (Intercept) | 32.082 | 1.226 | 26.159*** | | 13.576 | .552 | 24.579*** | 45.659 | 1.675 | | 27.253*** |  |
| Relationship = Distant | -5.448 | .661 | -8.239*** | | -2.565 | .298 | -8.611*** | -8.013 | .903 | | -8.870*** |  |
| Cause = Violent | 2.563 | .834 | 3.071** | | 1.083 | .376 | 2.882** | 3.646 | 1.140 | | 3.198** |  |
| Gender = Male | -1.914 | .629 | -3.04** | | -1.242 | .283 | -4.38*** | -3.155 | .860 | | -3.670*** |  |
| Financial Difficulties = Great | 4.659 | 1.230 | 3.787*** | | .937 | .554 | 1.69 | 5.596 | 1.681 | | 3.330*** |  |
| Sometimes | 1.847 | 1.157 | 1.597 | | .517 | .521 | .993 | 2.364 | 1.580 | | 1.496 |  |
| *Note. *** p < .001; ** p < .01; * p<.05;* **Standard**: SSA: F(5, 1487) = 26.27, p < .001), with R^2^ = .081 and adjusted R^2^ = .078. **Cultural**: SSA: F(5, 1487) = 24.31, p < .001, with R^2^ = .076 and adjusted R^2^ = .072. **Combined**: SSA: F(5, 1487) = 28.42, p < .001, with R^2^ = 0.087 and adjusted R^2^ = .084. | | | | | | | | | | | | |

**Supplementary Table 12 *List of ethical approval boards and numbers***


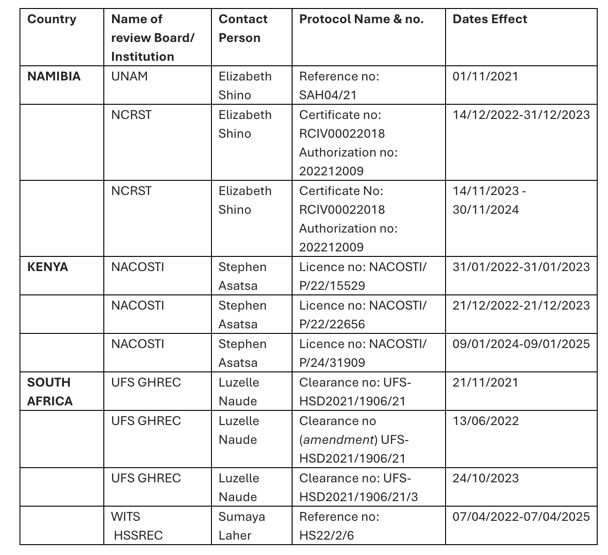


Mode of Administraion Analysis (online vs research assistant in person)

Independent samples Welch’s t-tests were conducted to examine whether mode of survey administration (online vs. through a research assistant) was associated with differences in International Prolonged Grief disorder scale (IPGDS) scores, including the standard symptom subscale, the cultural symptom subscale, and the combined total score.

For the **total sample** (Kenya, Namibia, and South Africa), participants who completed the survey online reported significantly lower scores than those assessed by a research assistant in person for standard symptoms, t(1378.30) = −5.18, p < .001, 95% CI [−4.58, −2.07], M = 28.11 vs. M = 31.43; cultural symptoms, t(1410.90) = −4.35, p < .001, 95% CI [−1.82, −0.69], M = 11.28 vs. M = 12.54; and total symptoms, t(1389.80) = −5.21, p < .001, 95% CI [−6.31, −2.86], M = 39.39 vs. M = 43.97.

When analyses were stratified by country, results varied. In **South Africa**, no significant differences were found between administration modes, ps > .10. In **Namibia**, research assistant administration yielded significantly higher scores than online administration for standard symptoms, t(318.20) = −3.15, p = .002, 95% CI [−5.51, −1.28], M = 29.53 vs. M = 32.92, and total symptoms, t(311.76) = −2.80, p = .005, 95% CI [−7.18, −1.25], M = 41.58 vs. M = 45.79, but no significant difference emerged for cultural symptoms, p = .117. In **Kenya**, research assistant administration was associated with significantly higher scores across all three measures: standard symptoms, t(511.93) = −2.94, p = .003, 95% CI [−5.37, −1.07], M = 27.73 vs. M = 30.96; cultural symptoms, t(513.28) = −3.80, p < .001, 95% CI [−2.65, −0.85], M = 10.18 vs. M = 11.93; and total symptoms, t(518.08) = −3.37, p = .001, 95% CI [−7.87, −2.07], M = 37.91 vs. M = 42.89.
