# Supplementary material for: Prolonged grief in African contexts: Scale validation, prevalence rates and risk factors among young adults in Kenya, Namibia and South Africa
Source: Glob Ment Health (Camb). 2026 May 18;13:e114. doi: 10.1017/gmh.2026.10220 (PMC13244226; doi:10.1017/gmh.2026.10220)
Supplement: Peer review history

# Reviewer report: Review: Prolonged grief in African contexts: Scale validation, prevalence rates and risk factors among young adults in Kenya, Namibia and South Africa — R0/PR2

**DOI:** 10.1017/gmh.2026.10220.pr2

**Authors:** Alice Einloft Brunnet

**Affiliations:** Univ Paris Nanterre

Thank you for the opportunity to review the manuscript titled Prolonged Grief in African contexts: Scale validation, prevalence rates and risk factors among young adults in Kenya, Namibia, and South Africa. The authors aimed to explore the psychometric validity of the International Prolonged Grief Disorder Scale (IPGDS) among young adults in three African countries. They also examined the probable prevalence of PGD and associated factors.

This study draws on data from an underrepresented population and therefore makes a valuable contribution to the field of PGD research. I also commend the authors for their open science efforts, which add value to this work. Despite its strengths, I believe the manuscript requires some revisions before it is suitable for publication.

General Comments

• I recommend that the authors provide citations to support statements such as “individuals may experience higher rates of death early in life” and “the death of loved ones before old age is much more common.” Are there studies on youth mortality rates in the included African countries that could be cited here?

• I encourage the authors to use more nuanced language when referring to this study as “the first” of its kind. Phrases like “to our knowledge” or “as far as we are aware” would be more appropriate and cautious.

Introduction

• I do not fully agree with the sentence: “Presently there are no clear guidelines for how to assess the relevant cultural norms…” For example, the Cultural Formulation Interview (CFI, DSM-5) provides a structured approach to assessing cultural aspects in clinical or research settings.

• The introduction would benefit from a brief discussion of other available prolonged grief instruments (e.g., PG-13, TGI-SR+), and a rationale for choosing to validate a new scale when others are already widely validated internationally.

Method

• The authors mention that participants speak over 50 different languages. In what language was the IPGDS administered? If the scale was translated, please describe the translation process (e.g., forward and back translation, cultural adaptation procedures). If the scale was only administered in English, the limitations of this choice could be acknowledged in the Discussion section.

• Table 6 could be moved to the supplementary materials.

• The definition of a “close” relationship could be included in the main text, not only in the supplementary materials.

• There may be a typographical error in the supplemental material regarding relationship categorization: “The type of relationship was also recoded, with ‘Close’ relationships encompassing spouse or romantic partner, child, sibling, and parent, and ‘Distant’ relationships including spouse or romantic partner, grandparent, and other relationships.” Here, “spouse or romantic partner” appears in both categories—please clarify.

• If I understood correctly, the analyses included participants who had lost a loved one less than six months ago. As mentioned on pages 9–10 (lines 58–5), “those who were bereaved less than 6 months ago had higher grief severity scores on the IPGDS.” Time since loss is a key diagnostic criterion for PGD. Including these participants could bias your findings, as you may be capturing normal grief reactions rather than PGD. I recommend excluding these cases at least from the prevalence and risk factor analyses.

Results

• It would be helpful to have more descriptive information about the socio-demographic characteristics of the sample. Variables such as income, urban vs. rural residence, and education level would help the reader to better understand the sample. If this information is not available, I suggest at least providing descriptive statistics for all variables used in the regression analyses. For example, “financial difficulties” appears in the regression model but is not described in the Participants section.

• Table notes should define all acronyms, even if they are explained in the main text.

Discussion

• The absence of a validated PGD measure (e.g., TGI-SR or PG-13) for use in convergent validity analysis could be acknowledged as a limitation.

# Reviewer report: Review: Prolonged grief in African contexts: Scale validation, prevalence rates and risk factors among young adults in Kenya, Namibia and South Africa — R0/PR3

**DOI:** 10.1017/gmh.2026.10220.pr3

This manuscript addresses an important and innovative topic, examining the psychometric properties of the IPGDS and the prevalence of PGD in young adults across multiple African countries. The study fills a significant gap in the literature and is generally well-written and methodologically solid. However, several conceptual, methodological, and interpretive issues require clarification and expansion to strengthen the manuscript and ensure its contributions are fully realized before publication.

Conceptual framing and rationale

1.1. The authors should provide a stronger rationale for focusing on probable PGD rather than formal diagnosis. The use of the term “probable diagnosis” is potentially confusing, as it is often applied to sub-threshold disorders and screening outcomes rather than survey-based estimates. Redefining this and its implications for interpreting the findings is essential.

1.2. The global context for this research seems somewhat underdeveloped. The introduction briefly mentions higher early-life exposure to death but does not sufficiently explain why validating the IPGDS in African contexts is particularly important. Please elaborate on the cultural, structural, and contextual factors that make this work critical, and compare with other non-Western populations (e.g., refugee samples) to underscore the relevance.

1.3. The statement that death of loved ones before old age is more common in African countries lacks supporting evidence or explanation. Providing empirical references or further elaboration is necessary to justify this claim.

1.4. Several arguments (e.g., predictors such as closeness to the deceased and violent death, Western vs. non-Western prevalence, risk factors) closely mirror findings from Hilberdink et al., 2023 (“Bereavement issues and prolonged grief disorder: A global perspective”) published in GMH. This review should be cited and integrated into the introduction and discussion to situate the current findings within existing literature.

1.5. In the third paragraph of the introduction, the inclusion of prevalence rates for adjustment disorder, PTSD, and complex PTSD is not clearly justified. Please clarify the purpose of presenting these disorders alongside PGD — is this intended as comparative context or for another reason?

1.6. The argument that lower prevalence rates may reflect protective factors seems overly narrow. Alternative explanations — such as cultural differences in symptom expression, measurement limitations, and the applicability of Western-based diagnostic criteria — should be explicitly discussed in both the introduction and discussion.

Prevalence and interpretation

2.1. The authors claim that prevalence rates are “similar to global averages.” This conclusion may be somewhat overstated. Please re-examine this statement and discuss whether the observed rates could actually be lower than global estimates, as well as provide a more nuanced interpretation of potential reasons for such differences.

2.2. The statement “Information on rates of trauma and PGD are urgently needed” should be expanded. Explain why such information is urgently needed (e.g., for informing clinical care, guiding cultural adaptation of tools, or shaping policy responses).

Study population

3.1. While the study focuses on young adults, comparable samples exist (e.g., Bryant et al., 2019, Epidemiol Psychiatry Sci, which included young adults in refugee populations). Please position this study more clearly within the existing literature.

Cultural context and adaptation

4.1. The paragraph on the role of ancestors in African cultural contexts is interesting but its relevance to PGD is unclear. Please explicitly link this cultural factor to grief processes and to the study’s aims.

4.2. The cultural supplement of the IPGDS was derived from interviews and validation in German and Chinese samples. Given the authors’ own emphasis on cultural distinctiveness, it would be important to discuss why these items are assumed to be relevant in African contexts or acknowledge the need for additional adaptation work.

Methods

5.1. The mode of administration varied across participants (e.g., survey vs. other formats), which could influence responses. Were there differences in scores based on administration mode? Please analyze or discuss this potential bias.

5.2. It is unclear why data were drawn primarily from Wave 4. Please clarify the rationale for prioritizing this wave and specify the role of other waves in the analyses.

5.3. Language of administration: Given the multilingual context (50+ languages), how was the IPGDS delivered? Were translations provided? Were participants able to complete the questionnaire in their native language? These details are essential for understanding measurement validity.

5.4. The diagnostic algorithm for PGD does not appear to account for the duration criterion (6–12 months post-loss). Yet the authors report that some participants experienced more recent losses. How were these cases handled in the algorithm? Given that grief severity was higher in those bereaved <6 months ago, this has major implications for interpreting official PGD rates according to guidelines.

5.5. The authors used the three “most relevant” IMHA subscales (depression, anxiety, PTSD), but the rationale for selecting these over others (e.g., substance abuse, anger) is not explained. Could the authors please clarify?

5.6. The diagnostic algorithm testing is described in a complex manner. A visualization or explicit mapping of IPGDS items to diagnostic criteria in the supplements would improve transparency.

5.7. Since the authors aim to provide a culturally robust measurement tool, additional validation analyses (e.g., test–retest reliability, item-level analyses) would strengthen the manuscript. Were these analyses possible with the existing dataset?

5.8. For the mediation analysis, clarify how PGD symptom severity at 6 and 18 months post-loss was measured. Was this assessed within the same participants or across different samples? This has implications for temporal interpretation and reliability (see comment 5.7).

Results

6.1. The finding that participants bereaved <6 months had higher grief severity (M = 34.8 vs. 29.9, p < .001) requires clarification: compared to whom?

6.2. The authors retained a unidimensional one-factor solution despite second eigenvalues >1 in some samples. Please provide a stronger justification for this decision, referencing theory and/or model fit indices.

6.3. For transparency, it would be important to report non-significant risk factors in addition to significant ones.

Discussion

7.1. The authors should comment on the secondary dimensions implied by eigenvalues >1. Were these explored or considered?

7.2. Differences between diagnostic algorithms across countries are important to discuss.

7.3. The third paragraph of the discussion is somewhat unclear and the authors should consider rewriting it for clarity.

7.4. The statement that a finding is “surprising given the high exposure to trauma in similar contexts” requires elaboration — why is it surprising?

7.5. There is inconsistent categorization of China as a Western vs. non-Western country. It would be helpful to standardize terminology across the manuscript.

7.6. When discussing protective factors, it would be important to also consider alternative explanations (e.g., overlap in Western/non-Western prevalence rates, measurement limitations, cultural differences in symptom presentation).

7.7. I would suggest the authors to discuss how the observed prevalence and risk factors compare with those found in African adult populations, as there may be meaningful overlaps.

Minor Revisions

- I would suggest the authors to proofread for typos and small inconsistencies.

- Reorganization of tables might be helpful. For example, moving the IPGDS table into the main manuscript and relocating the ethics table (currently Table 6) to the supplement.

- Missing statistics in tables should be added (e.g., R² for correlations, t-values for group differences) as well as a clarification of what values are presented in tables (means, percentages, SDs).

- It would be important to provide more specific references to supplementary materials (e.g., clarify the difference between Supplementary Table 11 and Table 5 in the manuscript).

Overall, this manuscript makes a meaningful contribution to the literature on PGD in young adults in African contexts. Addressing the conceptual framing, clarifying key methodological decisions, and providing more robust justification for psychometric choices would substantially strengthen the paper for publication.

# Editor report: Recommendation: Prolonged grief in African contexts: Scale validation, prevalence rates and risk factors among young adults in Kenya, Namibia and South Africa — R0/PR4

**DOI:** 10.1017/gmh.2026.10220.pr4

**Affiliations:** Primary Healthcare Sciences, University of Zimbabwe Faculty of Medicine, Zimbabwe

May you kindly address the major revisions as suggested by the reviewers.

# Editor report: Decision: Prolonged grief in African contexts: Scale validation, prevalence rates and risk factors among young adults in Kenya, Namibia and South Africa — R0/PR5

**DOI:** 10.1017/gmh.2026.10220.pr5

**Affiliations:** London School of Hygiene & Tropical Medicine, United Kingdom of Great Britain and Northern Ireland

No accompanying comment.

# Editor report: Recommendation: Prolonged grief in African contexts: Scale validation, prevalence rates and risk factors among young adults in Kenya, Namibia and South Africa — R1/PR7

**DOI:** 10.1017/gmh.2026.10220.pr7

**Affiliations:** Primary Healthcare Sciences, University of Zimbabwe Faculty of Medicine, Zimbabwe

Handling Editor Review Note

Manuscript: GMH-2025-0160.R1: Prolonged Grief in African contexts: Scale validation, prevalence rates and risk factors among young adults in Kenya, Namibia, and South Africa.

I have reviewed the reviewers’ comments and the authors’ response. Here are areas that require further clarification to improve the manuscript’s quality.

1. Introduction

Comment

The introduction would benefit from a brief discussion of other available prolonged grief instruments (e.g., PG-13, TGI-SR+), and a rationale for choosing to validate a new scale when others are already widely validated internationally.

Response

Thank you for this important point. We have now clarified this in the introduction and below.

Although previous validated assessment measures of PGD exist (i.e. PG-13 scale and the Traumatic Grief Inventory Self Report +) the International Prolonged grief disorder scale offers a unique assessment tool that balances the need for a universally reliable measure of PGD and the need to contextually assess cultural differences in grief symptoms. The scale contains two parts; one part standard ICD-11 items and the second part is a catalogue of cultural relevant symptoms.

Handling Editor’s Comment

The authors must provide a clear scientific rationale for the primary outcome measure. There is a need to clearly articulate the psychometric performance of the existing tools, for instance, what is the evidence of validity and reliability of the PG-13, and how is it comparable to other measures such as TGI-SR+. Importantly, what other feasibility parameters were considered in selecting the outcome measure? Currently, the authors just give a qualitative description of the tool, without a critical appraisal of the psychometrics and utility.

________________________________________

2. Language

Comment

The authors mention that participants speak over 50 different languages. In what language was the IPGDS administered? If the scale was translated, please describe the translation process (e.g., forward and back translation, cultural adaptation procedures). If the scale was only administered in English, the limitations of this choice could be acknowledged in the Discussion section.

Response

Thank you for this point. We have now clarified that the participants completed the surveys in English. This is the language in which all participants attended high school, and fluency in English was an inclusion criterion to complete survey. The participants however also had access to a local RA, who typically speaks their home language, in case they had questions.

Handling Editor’s Comment

May you kindly fully address the language bias? Also, it is not clear how the data collection was standardised if other participants completed in their native language with the assistance of a research assistant. There is a need to demonstrate that there was no measurement bias and that the data collection methods were equivalent.

________________________________________

3. Section 1.6

Comment

1.6. The argument that lower prevalence rates may reflect protective factors seems overly narrow. Alternative explanations — such as cultural differences in symptom expression, measurement limitations, and the applicability of Western-based diagnostic criteria — should be explicitly discussed in both the introduction and discussion.

Response

Thank you, please see revised introduction page 4:

While some studies suggest that lower observed prevalence rates of prolonged grief disorder (PGD) in certain populations may reflect protective factors, this explanation is overly narrow. Alternative factors, including cultural differences in grief expression, limitations of existing measurement tools, and the potential mismatch of Western-based diagnostic criteria with local mourning practices, may also contribute to these findings

Handling Editor’s Comment

Whilst the authors have responded to the reviewers’ comments, the discussion needs to be strengthened by citing other previous studies.

________________________________________

4. Section 5.2

Comment

5.2. It is unclear why data were drawn primarily from Wave 4. Please clarify the rationale for prioritizing this wave and specify the role of other waves in the analyses.

Response

This was a procedural choice. There are 8 waves in the large study however grief is only assessed once (at wave 4) to ensure that there are enough participants remaining in the study (attrition becomes higher with each wave) but also to ensure that participants were not over burdened with questionnaires. We only analyze the data from Wave 4 independently of the other waves.

Handling Editor’s Comment

May you include a sentence to this effect in the methods section so that its also very clear to prospective readers.

________________________________________

5. Section 5.7

Comment

5.7. Since the authors aim to provide a culturally robust measurement tool, additional validation analyses (e.g., test–retest reliability, item-level analyses) would strengthen the manuscript. Were these analyses possible with the existing dataset?

Response

Thank you for this important point. Unfortunately, this is beyond the scope of the current study however we may have access to follow up data on general mental health symptoms for a second manuscript.

Handling Editor’s Comment

May you formally acknowledge these eliminations in the manuscript and provide recommendations for additional work.

________________________________________

6. Section 6.2

Comment

6.2. The authors retained a unidimensional one-factor solution despite second eigenvalues >1 in some samples. Please provide a stronger justification for this decision, referencing theory and/or model fit indices.

Response

Thank you for this important point. We have now provided a clearer rationale for this decision.

Previous EFA studies have confirmed the predominantly unidimensional structure of the standard scale (core symptoms and associated grief items) (Killikelly., et al 2020). This is also replicated across different culture groups The cultural supplement however, often yields a multifactor solution (Killikelly et al.,2020).

Handling Editor’s Comment

The response would benefit from a stronger theoretical grounding. At present, it lacks robust theoretical arguments, which weakens its overall academic rigour. This issue is particularly important because the response does not adequately align with the theoretical underpinnings highlighted by the reviewers. Addressing this gap will help ensure that the argument is both coherent and well-supported within the relevant scholarly framework.

# Editor report: Decision: Prolonged grief in African contexts: Scale validation, prevalence rates and risk factors among young adults in Kenya, Namibia and South Africa — R1/PR8

**DOI:** 10.1017/gmh.2026.10220.pr8

**Affiliations:** London School of Hygiene & Tropical Medicine, United Kingdom of Great Britain and Northern Ireland

No accompanying comment.

# Editor report: Recommendation: Prolonged grief in African contexts: Scale validation, prevalence rates and risk factors among young adults in Kenya, Namibia and South Africa — R2/PR10

**DOI:** 10.1017/gmh.2026.10220.pr10

**Affiliations:** Primary Healthcare Sciences, University of Zimbabwe Faculty of Medicine, Zimbabwe

All comments have been sufficiently addressed.

# Editor report: Decision: Prolonged grief in African contexts: Scale validation, prevalence rates and risk factors among young adults in Kenya, Namibia and South Africa — R2/PR11

**DOI:** 10.1017/gmh.2026.10220.pr11

**Affiliations:** London School of Hygiene & Tropical Medicine, United Kingdom of Great Britain and Northern Ireland

No accompanying comment.
